# Fast-breathing vs chest-indrawing childhood pneumonia: baseline characteristics

**DOI:** 10.1016/j.ijid.2020.12.082

**Published:** 2021-03

**Authors:** Amy Sarah Ginsburg, Susanne May

**Affiliations:** University of Washington, Seattle, WA, USA

**Keywords:** Childhood pneumonia, Fast breathing, Chest indrawing, Africa

## Abstract

• Comparison of fast-breathing vs chest-indrawing community-acquired pneumonia.
• Male and younger children appear more vulnerable to chest-indrawing pneumonia.
• Understanding sex-, age-, and nutrition-related differences informs interventions.

In resource-constrained settings, World Health Organization (WHO) guidelines recommend using clinical symptoms and signs to classify and treat pediatric pneumonia (World Health Organization (WHO), 2014). Children with cough lasting <14 days or difficulty breathing and fast breathing for age or chest indrawing, in the absence of general or respiratory danger signs, are classified as having nonsevere pneumonia; what is unclear is whether aside from having fast breathing vs chest indrawing, do these children present differently? Identifying baseline characteristics that may be risk factors for more severe disease could aid in clinical decision-making.

Using data from two concurrent, independent, randomized clinical trials that evaluate amoxicillin treatment for nonsevere community-acquired pneumonia in children aged 2-59 months not infected with HIV in Malawi, (Ginsburg et al., 2019, Ginsburg et al., 2020) we compare baseline characteristics of children enrolled with fast-breathing pneumonia (FBP) vs chest-indrawing pneumonia (CIP). The trials were approved by the Western Institutional Review Board; College of Medicine Research and Ethics Committee; and Malawi Pharmacy, Medicines and Poisons Board; and were registered with ClinicalTrials.gov (Nos. NCT02760420 and NCT02678195).

The trials were conducted between June 2016 and April 2019 at Kamuzu Central Hospital and Bwaila District Hospital in Lilongwe, Malawi; a total of 1126 children with FBP and 3000 children with CIP were enrolled and included in this analysis (Table 1). The FBP trial included children with fast breathing for age as measured by using the respiratory rate assessment and no chest indrawing, while the CIP trial included children with visible indrawing of the chest wall with or without fast breathing as assessed by trained study physicians. FBP children were on average older than CIP children (mean ages of 21.3 vs 13.6 months and p-value <0.01). While 26.1% FBP children and 23.8% CIP children enrolled were aged 12-23 months, 34.8% FBP and 57.9% CIP children were aged <12 months. Children aged 2-5 years constituted 39.1% FBP as compared to 18.4% CIP. There was a slightly higher proportion of female FBP children than male (53.4% vs 46.6%), while the opposite was observed among CIP children (44.9% vs 55.1% and p-value <0.01). A higher proportion of CIP (4.5% with mid-upper arm circumference 115-125 mm) than FBP (1.4%) children had the evidence of moderate acute malnutrition (p-value <0.01); severe acute malnutrition was an exclusion criterion for both studies, though one FBP child with severe acute malnutrition was erroneously enrolled. Malaria rapid diagnostic test results were positive for a higher proportion of FBP than CIP children (12.5% vs 9.0% and p-value<0.01).

**Table 1:** Baseline characteristics of children enrolled in fast-breathing and chest-indrawing pneumonia clinical trials in Lilongwe, Malawi.

	Fast-breathing pneumonia clinical trial n = 1126	Chest-indrawing pneumonia clinical trial n = 3000	p-value
Age (months)			< 0.01^a^
2–6, n (%)	222 (19.7%)	1155 (38.5%)	
7 –11, n (%)	170 (15.1%)	581 (19.4%)	
12–23, n (%)	294 (26.1%)	713 (23.8%)	
24–35, n (%)	215 (19.1%)	310 (10.3%)	
36–59, n (%)	225 (20.0%)	241 (8.0%)	
Mean (SD)	21.3 (15.1)	13.6 (12.3)	
Sex			< 0.01^b^
Male, n (%)	525 (46.6%)	1653 (55.1%)	
Female, n (%)	601 (53.4%)	1347 (44.9%)	
Mid-upper arm circumference (mm)^c^			< 0.01^b^
>125	1109 (98.6%)	2864 (95.5%)	
115 - 125	16 (1.4%)	136 (4.5%)	
Malaria rapid diagnostic test			< 0.01^b^
Positive, n (%)	141 (12.5%)	269 (9.0%)	
Negative, n (%)	985 (87.5%)	2731 (91.0%)	
SD = standard deviation			

a Based on t-test.

b Based on chi-squared test or Fisher's exact test.

c Mid-upper arm circumference was missing for 1 child enrolled in the fast-breathing pneumonia 3-day amoxicillin arm.

When comparing children with community-acquired FBP vs CIP, male and younger children and those with the evidence of malnutrition appear to be more vulnerable to CIP and/or may more likely be brought in for care for other reasons. Male sex, younger age, and malnutrition have been shown to be associated with more severe disease presentations and increased mortality (le Roux et al., 2019). Understanding sex-, age-, and nutrition-related differences may inform the development of prevention or treatment interventions that can reduce the number of children who get and die from pneumonia. It was not surprising that more children in the FBP trial tested positive for malaria, given that fast breathing is also a clinical sign of malaria; a significant proportion of children diagnosed with pneumonia on the basis of having fast breathing in the setting of a cough or difficulty breathing may not have pneumonia and rather some other illness or process. In summary, identifying underlying vulnerabilities and characteristics in children presenting with community-acquired pneumonia may enable the identification of higher risk subgroups that require more intense care and monitoring.

## Funding

This work was funded by a grant from the Bill and Melinda Gates Foundation (OPP1105080).
